# Allometric patterns in *Rhamphorhynchus muensteri* and their role in the paleoecology, life habits, and taxonomic framework of pterosaurs

**DOI:** 10.1111/joa.70146

**Published:** 2026-05-06

**Authors:** Gabriela Menezes Cerqueira, João Lucas Da Silva, Ingmar Werneburg, Mario Bronzati, Alex Sandro Schiller Aires, Felipe L. Pinheiro, Rodrigo Temp Müller

**Affiliations:** ^1^ Centro de Apoio à Pesquisa Paleontológica da Quarta Colônia Universidade Federal de Santa Maria São João do Polêsine RS Brazil; ^2^ Programa de Pós‐Graduação Em Biodiversidade Animal Universidade Federal de Santa Maria Santa Maria RS Brazil; ^3^ Paläontologische Sammlung Senckenberg Centre for Human Evolution and Paleonevironment an der Universität Tübingen Tübingen Germany; ^4^ Laboratório de Paleobiologia Universidade Federal Do Pampa São Gabriel RS Brazil; ^5^ Fachbereich Geowissenschften Universität Tübingen Tübingen Germany; ^6^ Programa de Pós‐Graduação de Geociências, Instituto de Geociências Universidade Federal do Rio Grande do Sul Porto Alegre Brazil

**Keywords:** allometry, Euctenochasmatia, Eupterodactyloidea, evolution, isometry, Pterosauria, *Rhamphorhynchus muensteri*

## Abstract

Pterosaurs were the first vertebrates to achieve powered flight, a result of various unique anatomical adaptations, and significant morphological diversity throughout their evolutionary history. Although the pterosaurs body structure was specialized for powered flight, the clade exhibited great disparity from its origins, with later evolution giving rise to multiple phylogenetic lineages and a wide range of body sizes, from small forms such as *Anurognathus ammoni* to truly giant species like *Quetzalcoatlus northropi*. This variation led to distinct flight strategies within the group. Here, we assess allometry in the skeletal elements of *Rhamphorhynchus muensteri* in comparison with Eupterodactyloidea and Euctenochasmatia, examining their implications for paleoecology and flight capacity. The analysis included 127 specimens and assessed 13 skeletal variables using statistical methods, principal components analysis (PCA), and standardized major analysis (SMA). The results reveal differences in size–shape trajectories and functional adaptations related to activity flight. *R. muensteri* exhibits a trend of negative allometry (PCRW/body, humerus, radius/ulna, metacarpal IV, and femur), indicating distinct allometric scaling patterns and flight style. These patterns suggest behavior and ecological differences, as well differing flight capabilities between *R. muensteri* likely relied on active flapping flight interspersed with gliding, whereas other taxa may have exhibited greater migratory adaptations within Eupterodactyloidea, and a greater dependence on flapping flight in Euctenochasmatia. The negative allometry in *R. muensteri* suggests a flight style characterized by early flight capability (precocial flight), whereas Eupterodactyloidea showed positive allometry and Euctenochasmatia isometry. Additionally, the study highlights how allometric patterns influence pterosaur phylogeny, underscoring the importance of incorporating them into future analysis.

## Graphical abstract

The Graphical Abstract ilustrates the different allometric pattern within different Pterosauria groups, highlighting how the allometry influences the flight and habits of Pterosauria.

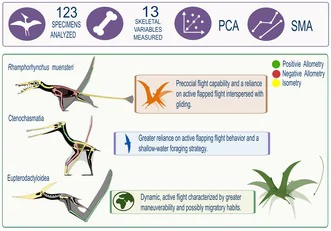

## 1 INTRODUCTION

Pterosauria was a clade of flying archosaurs that thrived from the Middle Triassic (according to ghost lineages) to the end of Cretaceous Period (approximately 245 to 125 Myr; Barrett et al., 2008; Butler et al., 2008, 2012, 2013; Ezcurra et al., 2020). They were the first vertebrates to evolve active flight, which was the result of a series of unique anatomical adaptations (Barrett et al., 2008; Britt et al., 2018; Ezcurra et al., 2020; McGowan & Dyke, 2007; Romer, 1966; Unwin, 2005; Wellnhofer, 1991; Witton, 2013). Although the skeletal structure of pterosaurs is somewhat conservative due to their adaptations to the powered flight, the clade achieved great morphological disparity from its origins (Müller et al., 2023), with its later evolution resulting in many phylogenetic lineages (Andres et al., 2010, 2014; Andres & Langston Jr, 2021; Bennett, 1989; Howse, 1986; Kellner, 2003; Unwin, 2003). Pterosaur morphological disparity can be exemplified by a wide variety of feeding apparatuses, cranial crests, dentition patterns, and body size, ranging from small animals such as *Pterodactylus antiquus*, Sömmerring, 1812, to exceptionally large animals such as *Quetzalcoatlus northropi*, Lawson, (1975). While pterosaur cranial skeleton displays considerable disparity, suggesting a diversity of behaviors, and feeding strategies (e.g., piscivore, collusive behavior, filter feeding, insectivore, aerial predation, frugivory, stalking, stork‐like foraging, and scavenging) (Bennett, 2007, 2013; Foth et al., 2012; Lü et al., 2010; Meyer & Hunt, 1999; Ősi et al., 2005; Padian et al., 2021; Unwin, 2005; Wellnhofer, 1991; Zhou et al., 2017), their general postcranial structure remained comparatively conservative throughout the Mesozoic Era, with few exceptions, such as anurognathids (*Jeholopterus ninchengensis*, Wang et al., 2012; and *Anurognathus ammoni*, Döderlein, 1923).

It has been shown for many extant and extinct groups that changes in timing and developmental scaling relationships in organisms are one of the main factors triggering macroevolutionary changes (Dodson, 1975; Gould, 1977; McKinney, 1988; Smith, 2001; Butler et al., 2009; Bhullar et al., 2012; Koyabu et al., 2014; Benson et al., 2014; Godoy et al., 2018; Schlindwein et al., 2022; Nojiri et al., 2023). This process (i.e., heterochrony) has as an ontogenetic basis, where size and shape of structures change during development, having allometry as a fundamental condition in embryonic development (Gould, 1977; McKinney, 1988; Pélabon et al., 2014; Smith, 2001, 2003). Different pterosaur clades have relatively well‐known ontogenetic series, with specimens of different developmental stages preserved in *Konservat‐Lagerstätten* worldwide, mostly in Germany, China, and Brazil (Bennett, 1993, 2007; Elgin & Frey, 2011; Hone et al., 2021; Lü et al., 2013; Manzig et al., 2014; Pêgas, 2025; Pinheiro & Rodrigues, 2017; Unwin & Martill, 2007; Wang et al., 2014). This makes Pterosauria a suitable clade for assessing macroevolutionary processes that were related to developmental phenomena. Although Pterosauria comprises a monophyletic group, the internal phylogenetic relationships of the clade are not entirely resolved, with many competing hypotheses regarding in‐group relationships (Andres et al., 2010, 2014; Kellner, 2003; Lü et al., 2008; Unwin, 2003; Wei et al., 2021).

Pterosaurs have classically been divided into two major groups: Pterodactyloidea and “Rhamphorhynchoidea,” a grade composed of several early‐diverging lineages (Andres et al., 2010, 2014; Andres & Langston Jr, 2021; Kellner, 2003; Lü et al., 2008; Unwin, 2003; Wei et al., 2021). The main morphological differences between these groups include the derived short tail and the long metacarpal IV in pterodactyloids (e.g., *Pteranodon longiceps*, Marsh, 1876), whereas the “rhamphorhynchoids” retain the plesiomorphic condition of a relatively long tail and short metacarpal IV (e.g., *Rhamphorhynchus meunsteri*, Meyer, 1846), although transitional forms are known, such as *Skiphosoura bavarica* (Andres et al., 2014; Hone et al., 2024; Lü, 2009; Padian, 2004; Plieninger, 1901; Unwin, 2005). Ontogenetic investigations are relatively common for Pterodactyloidea, particularly through histological studies, but remain scarce for non‐pterodactyloids (Araújo et al., 2025; Bennett, 2018; de Ricqlés et al., 2000; Duque et al., 2022; Hone et al., 2021; Kellner, 2015; Manzig et al., 2014; Prondvai et al., 2012; Vidovic & Martill, 2014; Yang et al., 2023). In particular, the effects of allometry and heterochrony on the body structure of these animals from a macroevolutionary perspective remain poorly understood. One of the largest and best‐known non‐ptrodactyloid pterosaurs is *Rhamphorhynchus meunsteri*, from the Late Jurassic Solnhofen *Lagerstätte* in southern Germany (Wellnhofer, 1975). This species is represented by a wide range of exceptionally preserved specimens spanning various developmental stages, from juveniles to large adults (Bennett, 1995; Hone et al., 2021; Wellnhofer, 1975). Many specimens are nearly complete and preserve soft‐tissue impressions such as wing membranes, tail vanes, and beaks (Hone et al., 2013; Hone & McDavid, 2025; Witmer et al., 2003). These features establish *R. muensteri* as a model organism for pterosaur research, making it one of the best‐studied species, and supported by one of the richest fossil records among Pterosauria.

Here, we examine the effects of allometry in *R. muensteri*. Our analyses aim to determine the effects of allometric patterns in body proportions of the taxon as well as to investigate the consequences of these macroevolutionary processes on the habits and behaviors of these animals. To analyze allometry, we utilized a diverse range of pterosaurs from two different clades, Eupterodactyloidea and Euctenochasmatia, representing various species and families as a comparison for this analysis, along with extensive sample of *R. muensteri* specimens, which comprises one of the most abundant and well‐preserved material within Pterosauria, with numerous nearly complete specimens documented (e.g., Gross, 1937; Hone et al., 2015; Ősi & Prondvai, 2009; Tischlinger & Frey, 2002; Wellnhofer, 1975).

## 2 MATERIALS AND METHODS

An allometric analysis was performed to verify the allometric signals of pterosaur skeletal elements following a protocol modified from Yang et al. (2022) (see Data S1 for more details). By using a simplified model of the typical pterosaur body morphology, we were able to gather data on otherwise uninformative specimens due the missing data, while losing statistical power signal, a worthy trade‐off. Measurements of 13 key skeletal lengths were taken for the analysis, namely, (I) basal skull length (from the occipital to the tip of the rostrum); (II) neck; (III) PCRW/body (*präcaudale Rumpfwirbelsäule* or precaudal column of the trunk); (IV) humerus; (V) radius/ulna; (VI) metacarpal IV; (VII–X) wing phalanx I–IV; (XI) tail; (XII) femur; and (XIII) tibia/tarsus (Figure 1). The analysis included 127 specimens of different genera and suprageneric Eupterodactyloidea taxa (*N* = 28) corresponding to *Sinopterus, Pteranodon, Zheiangopterus longirostris, Tupandactylus navigans*, and *Ikrandraco avatar*; Ectenochasmatia taxa (*N* = 18) corresponding to *Diopecephalus kochi, Pterodactylus antiquus*, and *Ctenochasma elegans*; and *Rhamphorhynchus muensteri* (Rhamphorhynchidae) specimens (*N* = 81) (see Data S1 for more details) (Figure 2). For Eupterodactyloidea, correlations of nine combinations were analyzed. Due to the frequent absence of sufficiently preserved neck, PCRW/body, and tail elements in the fossil material, these were excluded from the principal components analysis (PCA) and standardized major analysis (SMA) to avoid potential biases. For Euctenochasmatia, 11 combinations of skeletal measurement correlations were examined. Given the limited number of specimens preserving the tail, this element was excluded, following the same criteria adopted for Eupterodactyloidea. In contrast, for *R. muensteri*, 12 combinations were analyzed. This taxon preserves a sufficient number of specimens with all skeletal elements considered, and therefore, no elements were excluded from the analysis (Tables 1, 2, 3).

**FIGURE 1:**
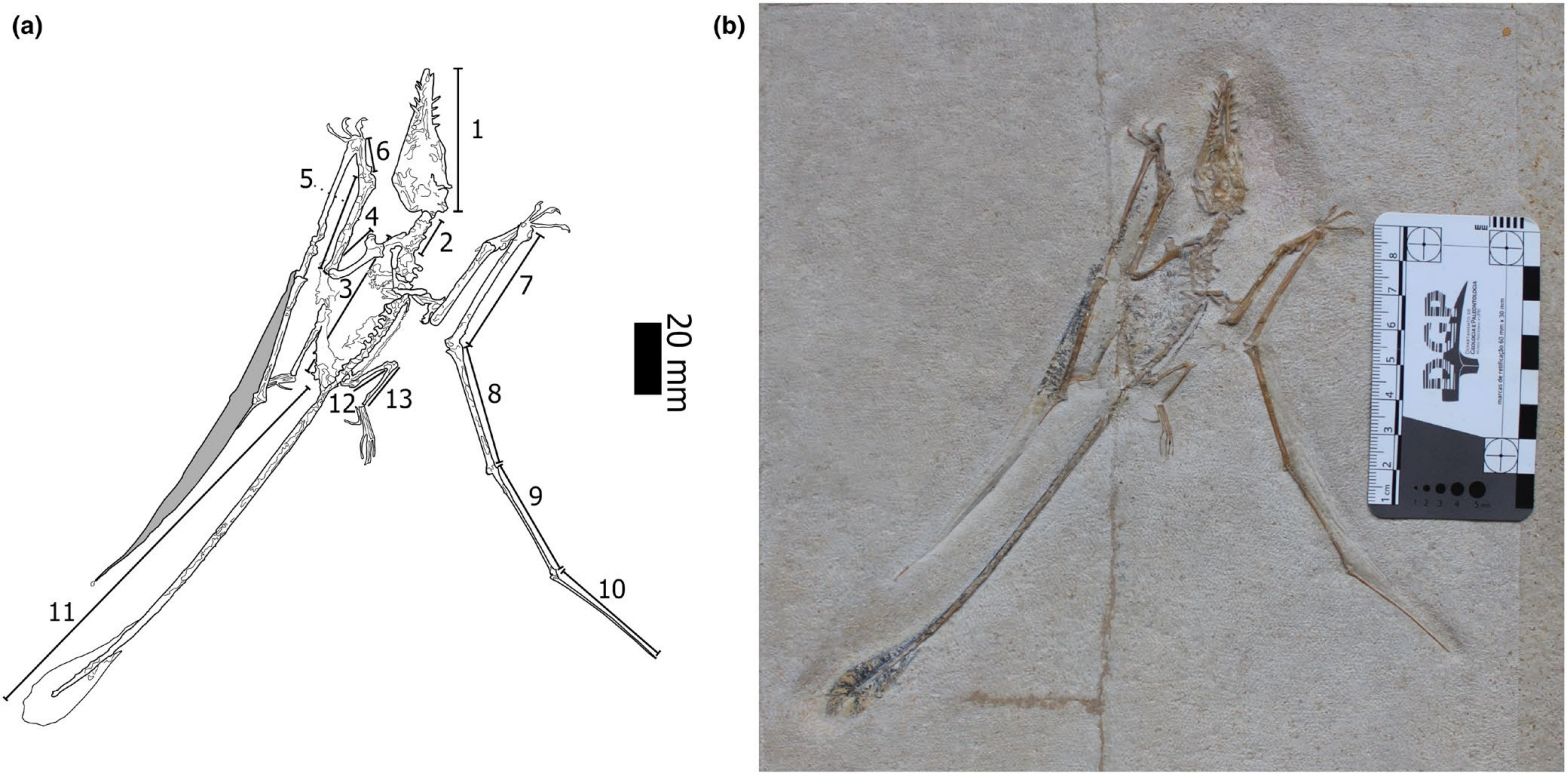
Line drawing of a generalized *Rhamphorhynchus* showing the locations where the measurements were taken. (a) Schematic drawing; (b) SNSB‐BSPG 1938 I 503/1. The numbers indicate the skeletal element: 1, skull; 2, neck; 3, PCRW/body; 4, humerus; 5, radius/ulna; 6, metacarpal IV; 7, wing phalanx I; 8, wing phalanx II; 9, wing phalanx III; 10, wing phalanx IV; 11, tail; 12, femur; and 13, tibia/tarsus. Scale of 20 mm.

**FIGURE 2:**
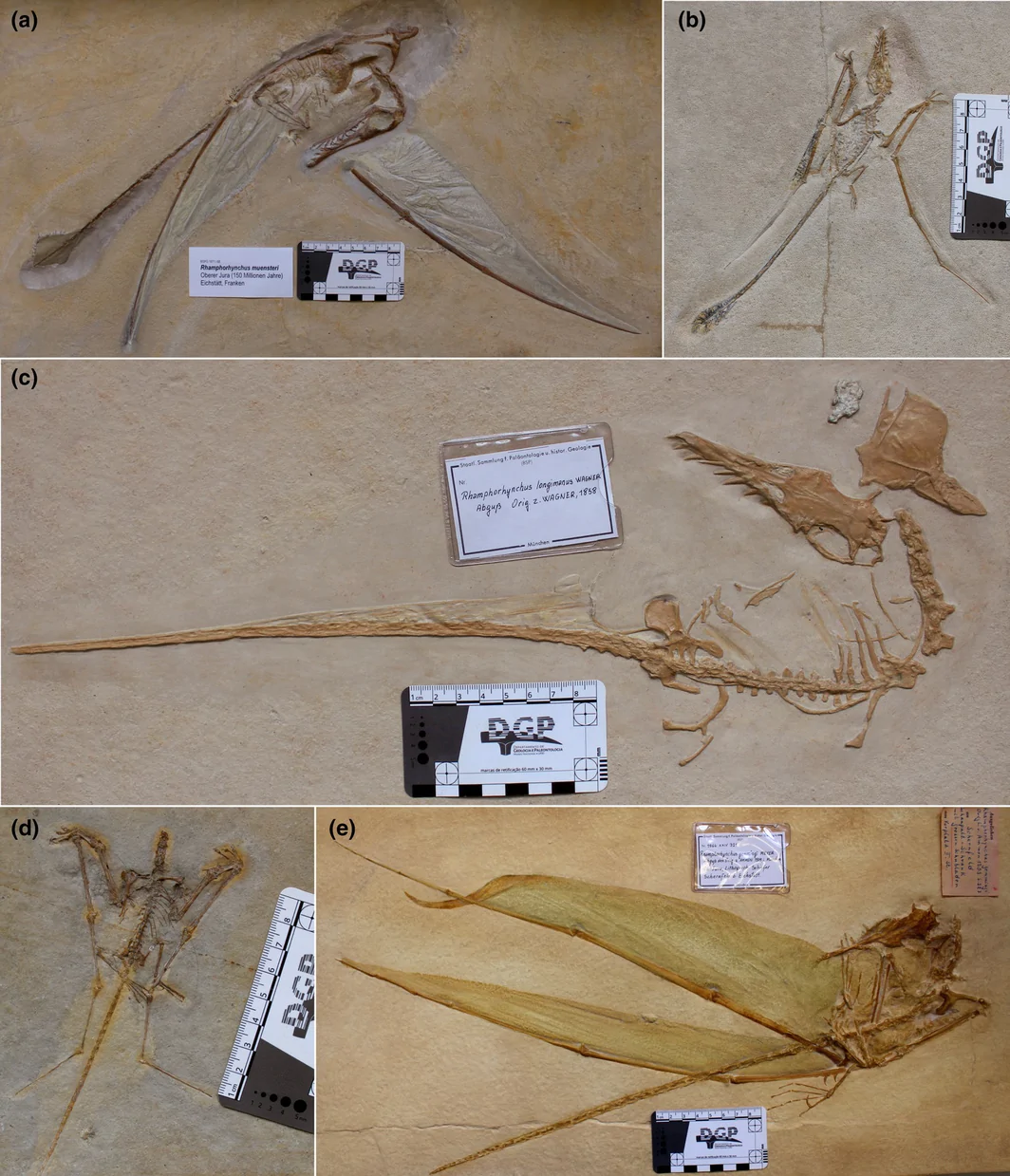
Photograph of specimens of *Rhamphorhynchus muensteri* utilized in the allometric analysis. (a) SNSB‐BSPG 1971 I 68; (b) SNSB‐BSPG 1938 I 503/1; (c) *Rhamphorhynchus longimanus* Wagner, 1858; (d) SNSB‐BSPG 1877 X 1; (e) SNSB‐BSPG 1964 XXIV 321. Scale of 80 mm.

**TABLE 1:** Allometric analysis referring to Eupterodactyloidea.

X	Y	*R* ^2^	*p*‐value	*a*	*b*	CI	Allometry
Radius/Ulna	Skull	0.92	<0.004	0.37	0.95	0.74–1.23	=
Radius/Ulna	Humerus	0.95	<0.002	−0.31	1.06	0.97–1.17	=
Radius/Ulna	McIV	0.94	<0.002	−0.51	1.27	1.16–1.40	+
Radius/Ulna	Wing phalanx I	0.95	<0.002	−0.24	1.19	1.08–1.31	+
Radius/Ulna	Wing phalanx II	0.97	<0.002	−0.37	1.20	1.11–1.30	+
Radius/Ulna	Wing phalanx III	0.95	<0.001	−0.50	1.19	1.07–1.34	+
Radius/Ulna	Wing phalanx IV	0.83	<0.001	−0.95	1.27	1.02–1.58	+
Radius/Ulna	Femur	0.96	<0.002	−0.02	0.96	0.87–1.07	=
Radius/Ulna	Tibia/Tarsus	0.94	<0.002	0.33	0.86	0.76–0.99	−

Abbreviations: CI, confidence interval; McIV, metacarpal IV.

The measurements were obtained from direct access to specimens, literature, and photographs. Those measurements taken from photographs were acquired using the freeware MB‐Ruler (http://www.markus‐bader.de/MB‐Ruler/index.php), a software that allows measuring angles and lengths from scaled photographs. Specimens were then subjected to principal component analysis (PCA), to identify the best proxy for use in the bivariate analysis and to evaluate whether humerus length (often considered a reliable proxy for body size) is indeed suitable as the primary proxy for body size and body mass, given that pterosaurs were forelimb‐dominated launchers (Habib, 2008; Pêgas et al., 2023; Wang et al., 2017; Wellnhofer, 1970, 1975; Yang et al., 2022). However, if another element proved to be a better proxy for a given group, the element explaining the greatest variance was used instead of humeral length.

For the PCA, the skeletal variables were not log‐transformed. Instead, the data were standardized (mean = 0, standard deviation = 1) to improve the compatibility of allometric coefficients. The first and second components (PC1, PC2) were extracted as internally defined size variables. The missing data were imputed using the iterative PCA method (Kiers, 1997), also known as the EM‐PCA algorithm (Josse & Husson, 2012), following the protocol of Yang et al. (2022). The analyses were performed in the R programming environment (R Core Team, 2023) with assistance of the packages “missMDA v1.18” (Josse & Husson, 2016) and “boot v1.3.28.1” (Canty & Ripley, 2016). The package “factoextra v.0.7” (Kassambara, 2016) was utilized to extract the maximum variance explained by each principal component (PC).

After the PCA, a bivariate analysis was performed to verify the allometric signals of the skeletal elements, using the standardized major analysis (SMA) following a modified protocol from Yang et al. (2022). The bivariate analysis (SMA) was performed to verify the correlation between size and shape variation and the potential allometric relationship among variables. This analysis was applied to determine allometric signals, coefficients of determination (*R* ^2^), and the *p*‐value to verify the statistical significance of correlations of each skeletal element pair tested (Cameron & Windmeijer, 1997). Bones with no available data of skeletal length were not included in the analysis. All values were log‐transformed for the analysis.

The analyses were performed in the RStudio with assistance of the packages “smart v3.4.8” (Warton et al., 2012), “dplyr v1.1.2” (Wickham et al., 2018), and “purr v1.0.1” (Henry & Wickham, 2020) for the SMA analysis, as in Yang et al. (2022). The *p*‐values were calculated to test the correlation between body size and each analyzed skeletal variable. When statistical significance was confirmed, an analysis of allometric relationship was performed using linear regression (ordinary least squares—OLS). Isometry is considered the null hypothesis for the analysis. The analyses of the variables with the appropriate proxy were performed to determine whether slopes were significant, considering the result of >1.0 as positive allometry, <1.0 as negative allometry, and results as 1.0 as isometry. In order to determine 95% confidence intervals (CI) of the SMA analysis, a bootstrap with 1.000 iterations was calculated for each SMA analysis performed for the skeletal variables, where the null hypothesis of isometry was rejected if the CI results stood entirely above or below 1.0 (e.g., 2±%), what indicates, respectively, positive and negative allometry (Canty & Ripley, 2016).

## 3 RESULTS

### 3.1 Principal components analysis

PC1 explains 93.4% of variation, whereas PC2 accounts for only 4.5% of the variation for Eupterodactyloidea. The variables contributing most to PC1 are the radius/ulna, humerus, wing phalanx II, and wing phalanx III, with the contributions of 10.6%, 10.5%, 10.5%, and 10.3%, respectively. For PC2, the variables that contribute most are the skull, metacarpal IV, wing phalanx I, and wing phalanx II contributing with 65.4%, 13.3%, 9.9%, and 2.8%, respectively (Figure 3) (see Supporting Information S2). For Euctenochasmatia, a total of 12 variables were used in the PCA, with PC1 explaining 89.5% of the variation, while PC2 explains 8.1%. Together, the PCs explain 97.6% of the total variation. The variables contributing most to PC1 are the wing phalanx II, tibia/tarsus, radius/ulna, and wing phalanx III contributing with 9.23%, 9.23%, 9.17%, and 9.13%, respectively. For PC2, the variables that contribute most are the PCRW/body, neck, skull, and femur contributing with 97.2%, 0.60%, 0.46%, and 0.38%, respectively (Figure 4) (see Supporting Information S2).

**FIGURE 3:**
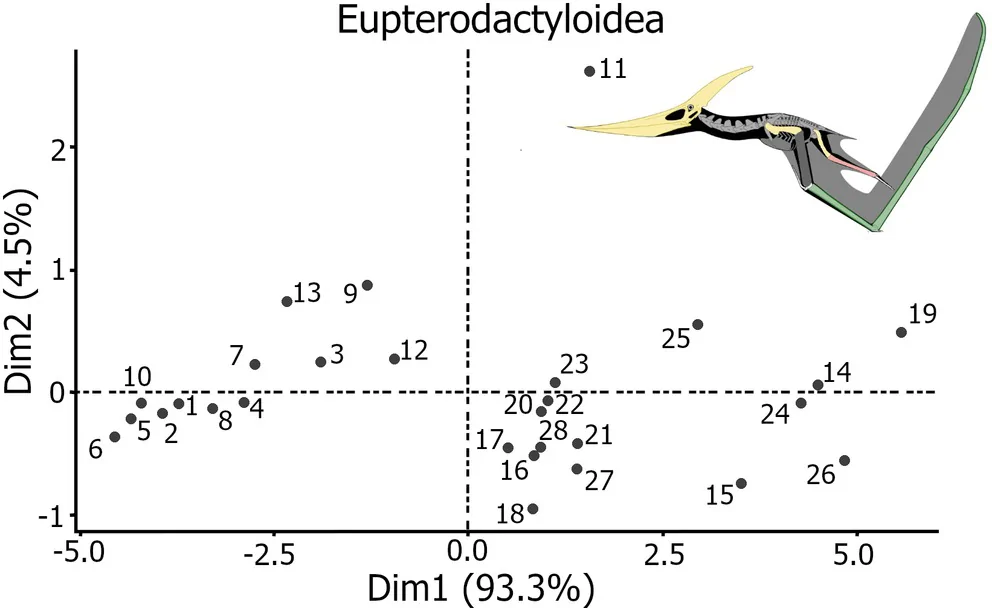
Principal component analysis of the Eupterodactyloidea individuals and their distribution and contribution on the factor map. The silhouette demonstrates the allometric signal of Eupterodactyloide. Red, negative allometry; green, positive allometry; yellow, isometry; dark gray, proxy used; and light gray, the variables not included in the analysis. The skeleton is based on the artwork by Scott Hartman.

**FIGURE 4:**
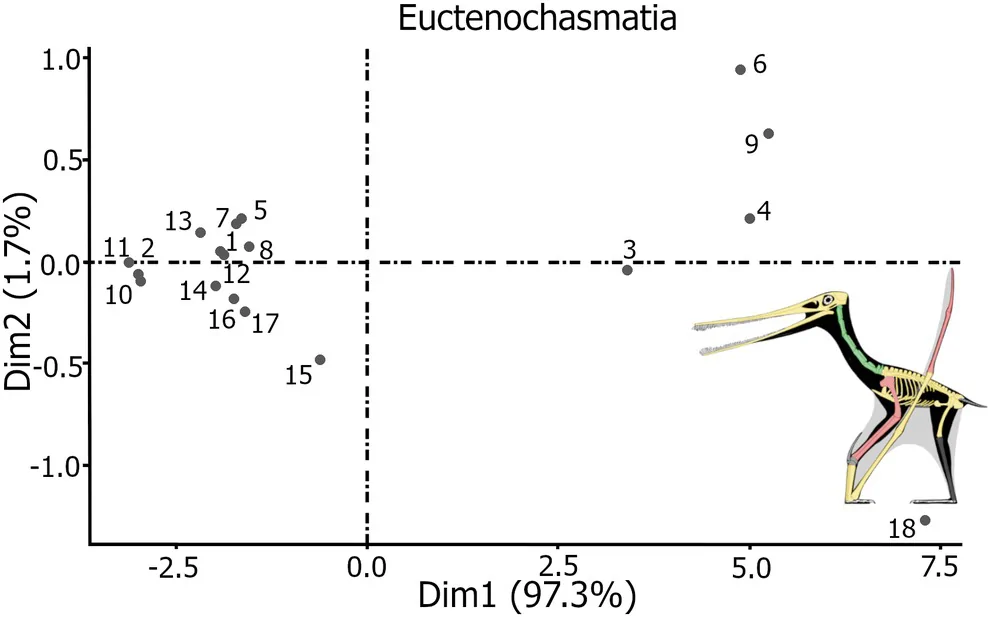
Principal component analysis of the Euctenochasmatia individuals and their distribution and contribution on the factor map. The silhouette demonstrates the allometric signal of Eupterodactyloide. Red, negative allometry; green, positive allometry; yellow, isometry; dark gray, proxy used; and light gray, the variables not included in the analysis. The skeleton is based on the artwork by Dean Schnabel.

Regarding the PCA analysis for *R. meunsteri*, PC1 explained 95.5% of the variation, whereas the PC2 accounted for only 1.3%, together explaining 96.6% of the total variation. The variables that contributed most to PC1 were wing phalanx I, wing phalanx II, radius/ulna, and tail, contributing with 7.93%, 7.88%, 7.85%, and 7.83% respectively. For PC2, the variables that contributed most were the humerus, metacarpal IV, wing phalanx III, and wing phalanx IV, contributing with 50.2%, 15.8%, 9.6%, and 7.7% respectively (Figure 5) (see Supporting Information S2).

**FIGURE 5:**
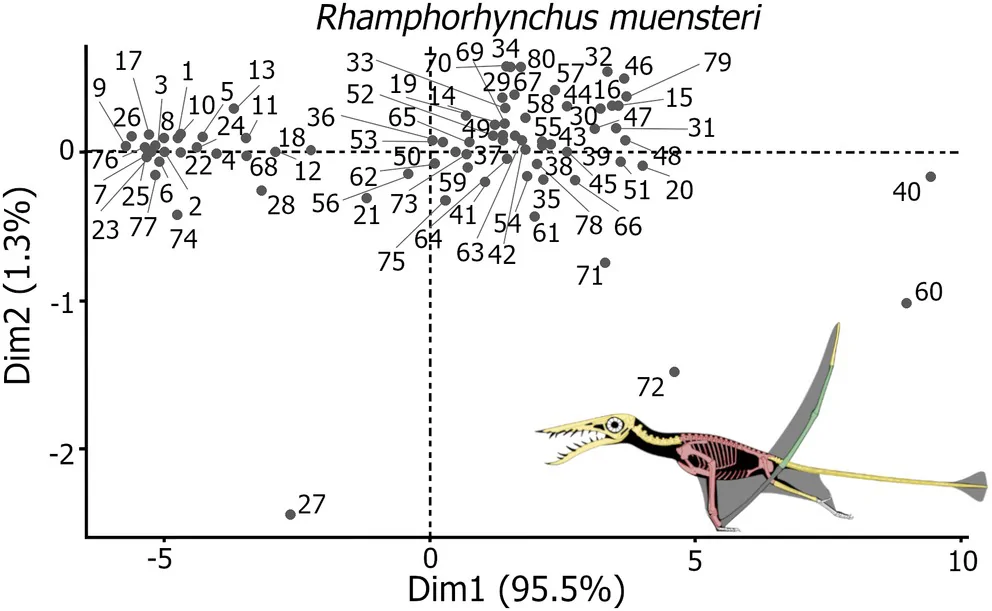
Principal component analysis of the *Rhamphorhynchus muensteri* individuals and their distribution and contribution on the factor map. The skeleton demonstrates the allometric signal of Eupterodactyloide. Red, negative allometry; green, positive allometry; yellow, isometry; dark gray, proxy used. The silhouette is based on the artwork by Scott Hartman.

### 3.2 Standardized major analysis

The bivariate analysis revealed significant correlations (*p* ≤ 0.005) across all three groups analyzed: Eupterodactyloidea, Euctenochasmatia, and *Rhamphorhynchus muensteri*. For Eupterodactyloidea, the radius/ulna showed a strong correlation with the other variables, with 33.3% of comparisons yielding 0.90 ≤ *R* ^2^ < 0.95, 55.5% yielding *R* ^2^ ≥ 0.95, and only 11.2% showing *R* ^2^ < 0.90. These results indicate that, despite the inclusion of multiple eupterodactyloid specimens, about 89% of the skeletal variables display conservative allometric scaling, with the notable exception of wing phalanx IV (Table 1). In Euctenochasmatia, wing phalanx II exhibited the strongest correlation with the other variables. Here, 36.4% of comparisons yielded 0.90 ≤ *R* ^2^ < 0.95, and 63.6% yielded *R* ^2^ ≥ 0.95. Demonstrating that the variables exhibit conservative allometric scaling (Table 2). In contrast, the analysis of *R. muensteri* showed 50% of comparisons with 0.90 ≤ *R* ^2^ < 0.95, 33.3% with *R* ^2^ ≥ 0.95, and 16.7% with *R* ^2^ < 0.90. These results indicate that about 83% of the variables exhibit conservative allometric scaling, with exceptions observed in the humerus and metacarpal IV (Table 3).

**TABLE 2:** Allometric analysis referring to Euctenochasmatia.

X	Y	*R* ^2^	*p*‐value	*a*	*b*	CI	Allometry
Tibia/Tarsus	Skull	0.96	<0.001	0.18	1.08	0.97–1.19	=
Tibia/Tarsus	Neck	0.96	<0.003	−0.05	1.13	1.01–1.27	+
Tibia/Tarsus	PCRW/body	0.91	<0.001	0.30	0.90	0.77–1.05	=
Tibia/Tarsus	Humerus	0.94	<0.006	0.18	0.78	0.68–0.88	−
Tibia/Tarsus	Radius/Ulna	0.98	<0.001	0.12	0.92	0.85–0.99	−
Tibia/Tarsus	McIV	0.95	<0.007	−0.10	1.00	0.90–1.12	=
Tibia/Tarsus	Wing phalanx I	0.95	<0.001	0.04	0.97	0.87–1.09	=
Tibia/Tarsus	Wing phalanx II	0.97	<0.002	0.03	0.96	0.88–1.05	=
Tibia/Tarsus	Wing phalanx III	0.94	<0.002	0.18	0.81	0.71–0.92	−
Tibia/Tarsus	Wing phalanx IV	0.93	<0.005	0.14	0.78	0.67–0.91	−
Tibia/Tarsus	Femur	0.97	<0.007	−0.08	0.95	0.87–1.05	=

Abbreviations: CI, confidence interval; McIV, metacarpal IV; PCRW/Body, precaudal trunk spine.

**TABLE 3:** Allometric analysis referring to *Rhamphorhynchus muensteri* specimens.

X	Y	*R* ^2^	*p*‐value	*a*	*b*	CI	Allometry
Wing phalange I	Skull	0.94	<0.002	−0.04	1.01	0.95–1.08	=
Wing phalange I	Neck	0.92	<0.002	−0.26	1.03	0.95–1.12	=
Wing phalange I	PCRW/body	0.93	<0.002	0.17	0.90	0.82–0.98	−
Wing phalange I	Humerus	0.88	<0.002	−0.07	0.82	0.76–0.89	−
Wing phalange I	Radius/Ulna	0.98	<0.002	0.11	0.84	0.81–0.87	−
Wing phalange I	McIV	0.88	<0.002	−0.12	0.71	0.66–0.78	−
Wing phalange I	Wing phalanx II	0.98	<0.002	−0.17	1.07	1.04–1.11	+
Wing phalange I	Wing phalanx III	0.96	<0.002	−0.25	1.09	1.04–1.14	+
Wing phalange I	Wing phalanx IV	0.94	<0.002	−0.05	0.99	0.93–1.05	=
Wing phalange I	Tail	0.93	<0.002	0.50	0.97	0.90–1.05	=
Wing phalange I	Femur	0.94	<0.002	−0.19	0.84	0.78–0.89	−
Wing phalange I	Tibia/Tarsus	0.95	<0.002	−0.36	1.00	0.94–1.05	=

Abbreviations: CI, confidence interval; McIV, metacarpal IV; PCRW/Body, precaudal trunk spine.

The SMA performed for Eupterodactyloidea, using the radius/ulna as a proxy for body size, revealed strong correlations for all elements analyzed (*R* ^2^ = 0.83–0.97, *p* < 0.004; Table 1). The skull, humerus, and femur exhibited isometric scaling, indicating that these elements scale proportionally with body size (Figures 6a,b and 7b). In contrast, metacarpal IV and wing phalanges I–IV displayed positive allometry, suggesting disproportionate elongation relative to body size (Figures 6c–f and 7a). The tibia/tarsus was the only element to exhibit negative allometry, indicating relatively reduced scaling compared to body size (Figure 7c). These results demonstrate that the most skeletal elements in Eupterodactyloidea scale predictably with body size variation, with forelimb elements particularly the wing phalanges undergoing marked elongation relative to body size.

**FIGURE 6:**
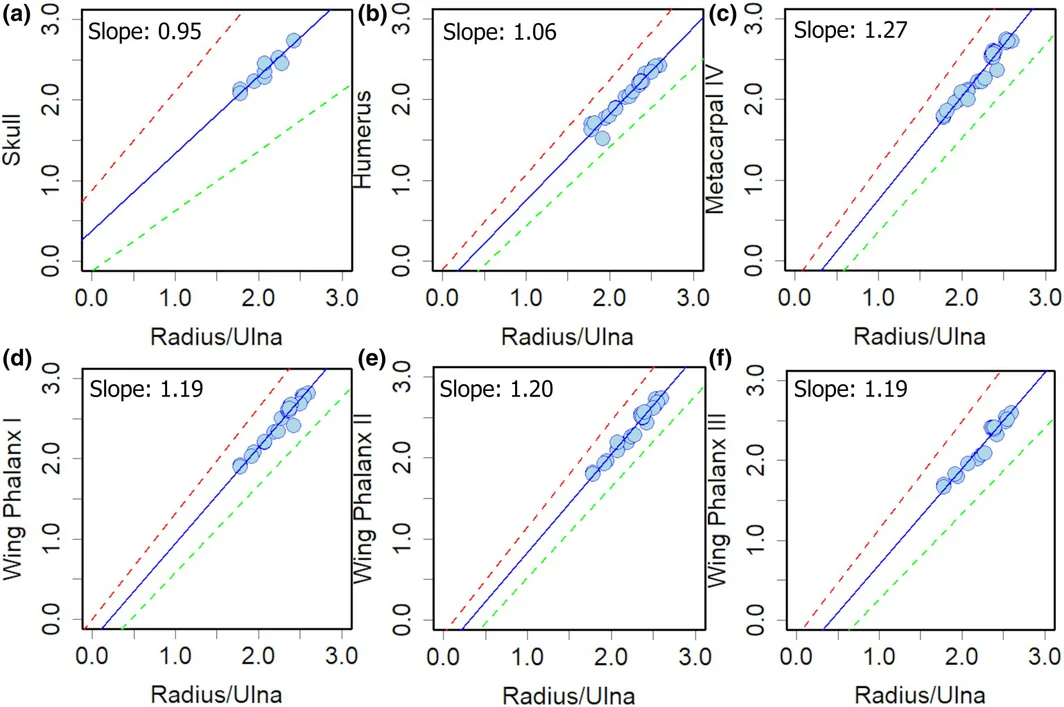
SMA plots of skeletal elements versus radius/ulna length in Eupterodactyloidea. (a) Skull. (b) Humerus. (c) Metacarpal IV. (d) Wing phalanx I. (e) Wing phalanx II. (f) Wing phalanx III.

**FIGURE 7:**
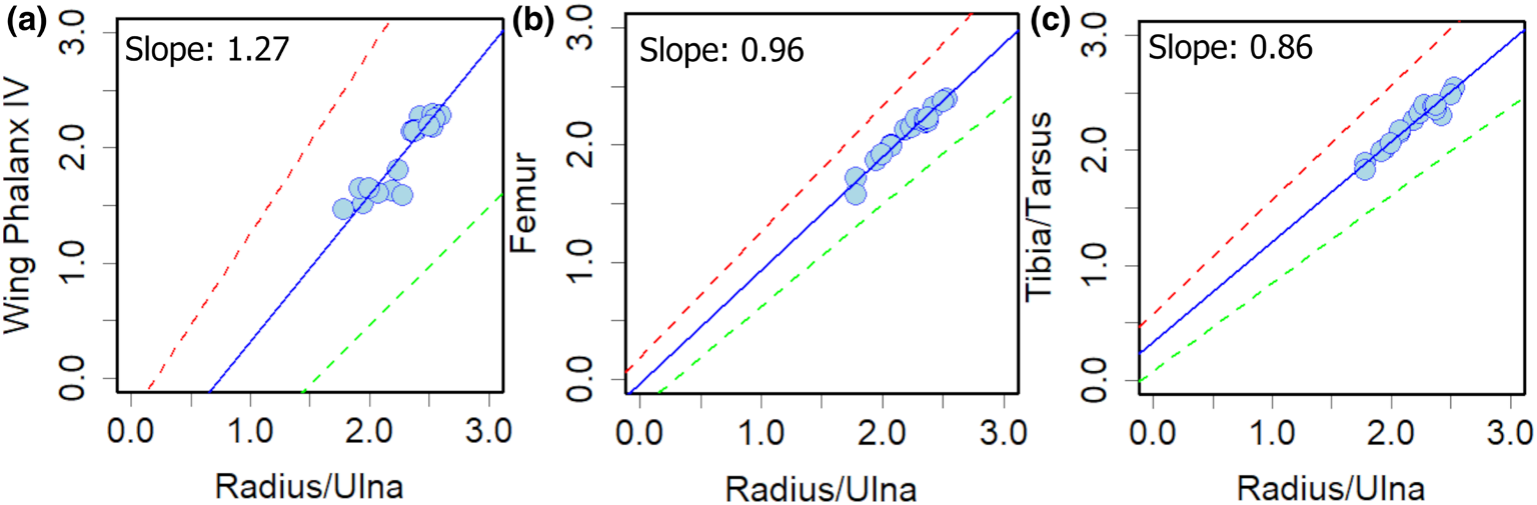
SMA plots of skeletal elements versus humerus length in Eupterodactyloidea. (a) Wing phalanx IV. (b) Femur. (c) Tibia/tarsus.

The SMA performed for Euctenochasmatia, using tibia/tarsus as a proxy for body size, revealed strong correlations for elements analyzed (*R* ^2^ = 0.93–0.98, *p* < 0.007), (Table 2). The skull, PCRW/body, metacarpal IV, wing phalanges I and II, and femur exhibited isometric scaling, indicating proportional scaling with body size (Figures 8a,c,f and 9a,b,e). In contrast, the neck displayed positive allometry, suggesting disproportionate elongation relative to body size (Figure 8b). The humerus, radius/ulna, and wing phalanx III–IV were the only elements to exhibit negative allometry, indicating relatively reduced scaling compared to body size (Figures 8d,e and 9c,d). These results indicate that, within Euctenochasmatia, most skeletal elements scale isometrically, with marked positive allometry restricted to the neck.

**FIGURE 8:**
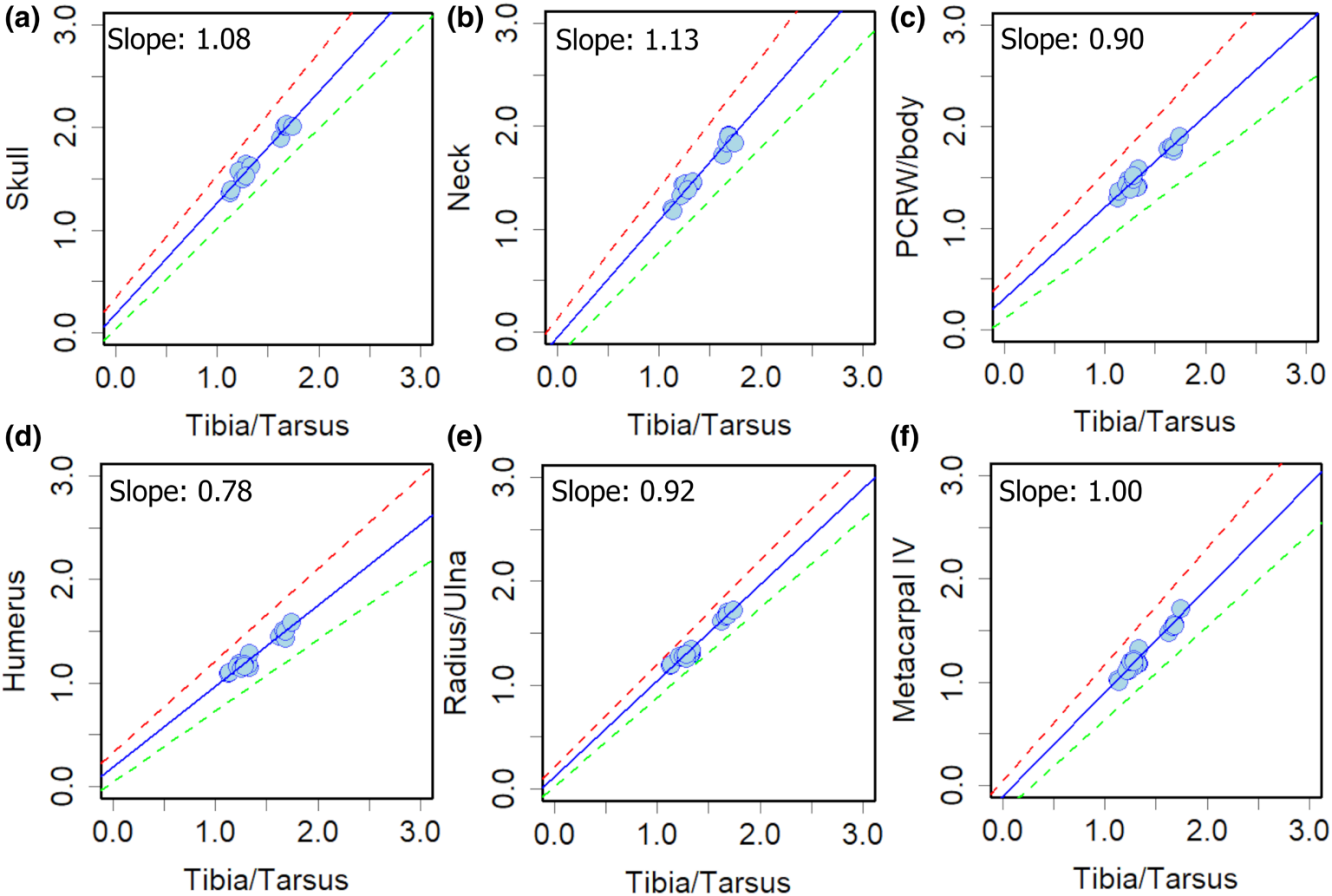
SMA plots of skeletal elements versus tibia/tarsus in Euctenochasmatia. (a) Skull. (b) Neck. (c) PCRW/body. (d) Humerus. (e) Radius/ulna. (f) Metacarpal IV. PCRW, precaudal trunk spine.

**FIGURE 9:**
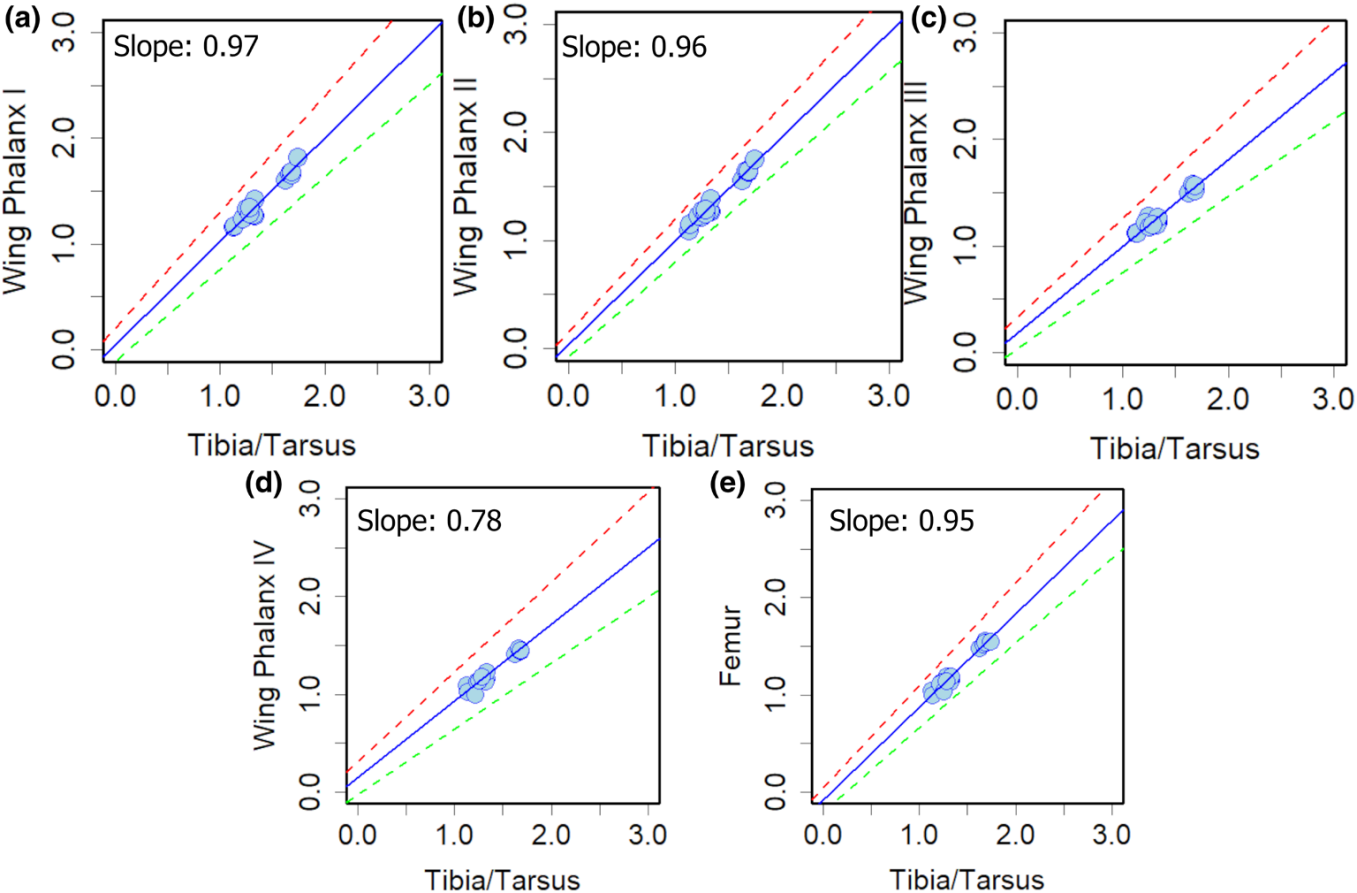
SMA plots of skeletal elements versus tibia/tarsus in Euctenochasmatia. (a) Wing phalanx I. (b) Wing phalanx III. (c) Wing phalanx IV. (d) Femur. (e) Tibia/tarsus.

The SMA performed for *Rhamphorhynchus muensteri*, using wing phalanx I as a proxy for body size, revealed strong correlations across all elements analyzed (*R* ^2^ = 0.88–0.98, *p* < 0.002; Table 3). The skull, neck, wing phalanx IV, tail, and tibia/tarsus exhibited isometric scaling, indicating proportional scaling with body size (Figures 10a,b and 11c,d,f). In contrast, wing phalanges II and III displayed positive allometry, suggesting disproportionate elongation relative to body size (Figure 11a,b). Negative allometry was observed in PCRW/body, humerus, radius/ulna, metacarpal IV, and femur, indicating relatively reduced scaling compared to overall body size (Figures 10c–f and 11e). Collectively, these results highlight that, while most elements in *R. muensteri* scale isometrically, the forelimb elements exhibit contrasting allometric patterns, with the wing phalanges elongating disproportionately whereas the proximal forelimb elements exhibit more conservative scaling relationships.

**FIGURE 10:**
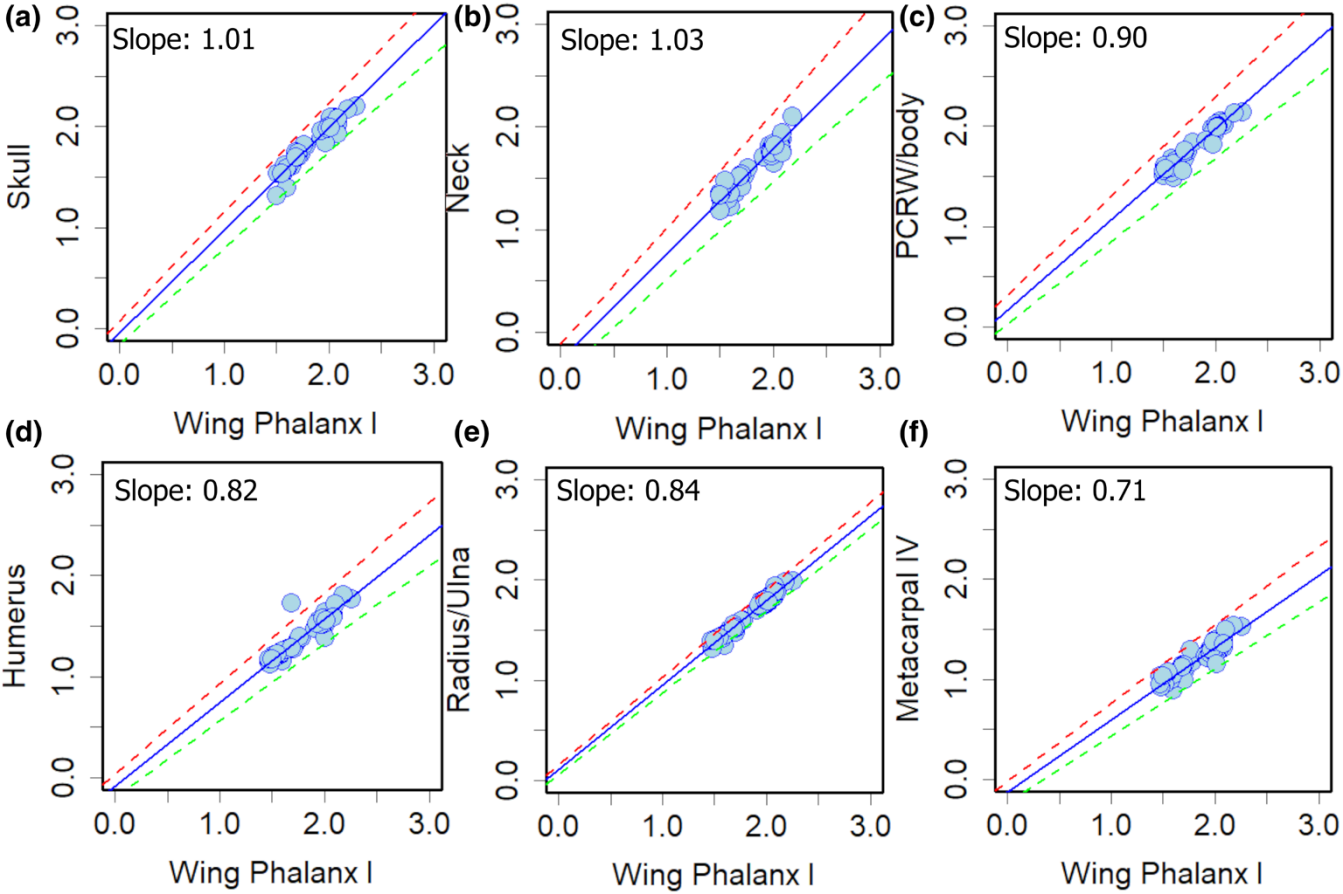
SMA plots of skeletal elements versus wing phalanx I in *Rhamphorhynchus muensteri*. (a) Skull. (b) Neck. (c) PCRW/body. (d) Humerus. (e) Radius/ulna. (f) Metacarpal IV. PCRW, precaudal trunk spine.

**FIGURE 11:**
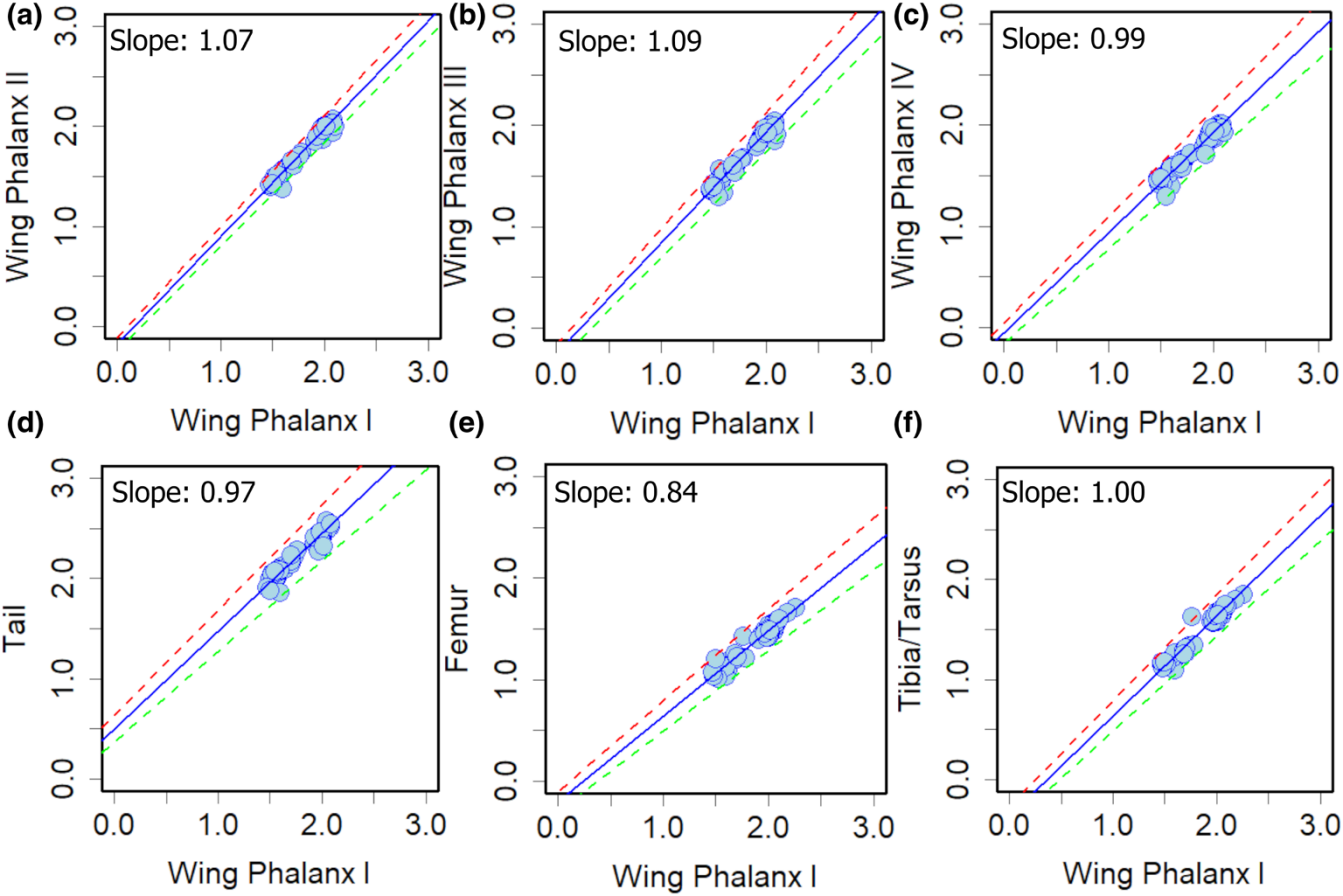
SMA plots of skeletal elements versus wing phalanx I in *Rhamphorhynchus muensteri*. (a) Wing phalanx II. (b) Wing phalanx III. (c) Wing phalanx IV. (d) Tail. (e) Femur. (f) Tibia/tarsus.

## 4 DISCUSSION

Based on the allometric analysis performed here, we tested the effects of allometry on the skeletal structure of a broad sample of Eupterodactyloidea, Euctenochasmatia, and *R. muensteri*. Our analysis revealed a distinct pattern of allometric scaling among the groups, which do not share the same growth trajectory (Figure 12a–c). Eupterodactyloids exhibit a trend to positive allometry on five wing elements. Isometry dominated the allometric trajectory of Euctenochasmatia. Negative allometry is overtly more common in *Rhamphorhynchus muensteri*, but restricted to trunk, proximal front limb elements, metacarpal IV, and femur.

**FIGURE 12:**
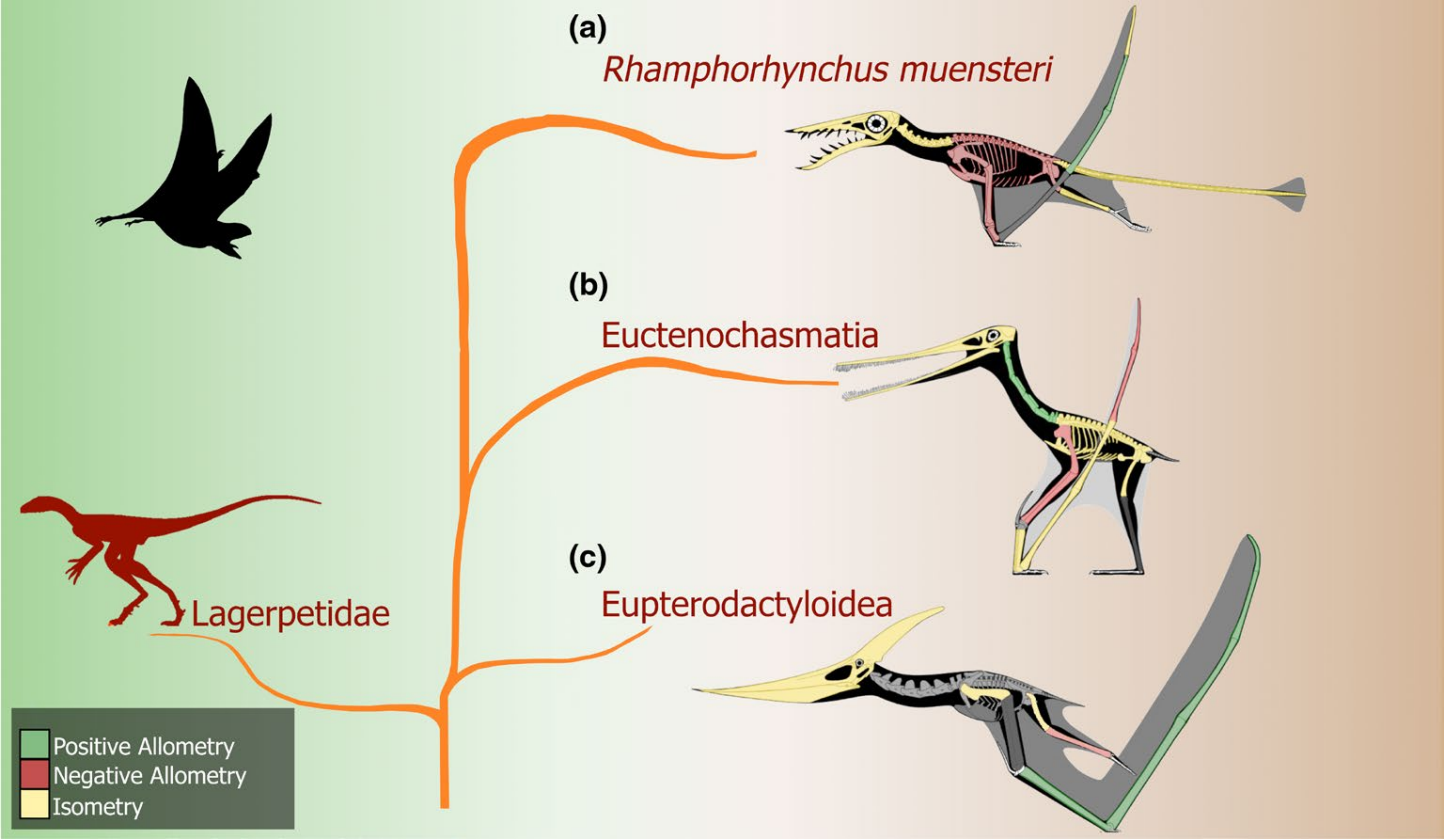
Growth allometry of elements in the studied group of pterosaurs. Red, negative allometry; green, positive allometry; yellow, isometry; and dark gray, proxy used; and light gray, the variables not included in the analysis. (a) *Rhamphorhynchus muensteri*, (b) Euctenochasmatia, and (c) Eupterodactyloidea. The silhouettes and skeletons are based on the artwork by Maurício Garcia, Dmitry Bogdanov, Dean Schnabel, and Scott Hartman.

Comparatively, these three groups display both convergent and divergent allometric patterns. Eupterodactyloidea and *Rhamphorhynchus muensteri* show a clear emphasis on distal wing element elongation, consistent with adaptations for flight, whereas Euctenochasmatia demonstrate a more modular approach, with selective elongation of the neck, consistent with ecological specialization. Isometric scaling in the skull is consistent across all groups, suggesting functional constraints that preserve essential biomechanical and cranial proportions.

Previous studies investigating changes in skeletal proportions during different size stages in individual pterosaurs have emphasized the need to reassess certain clades or genera, as such revisions are often crucial for distinguishing diagnostic characteristics of species that may actually represent different ontogenetic stages (Wellnhofer, 1970; Bennett, 1995, 1996; Bennett, 2006; Bennett, 2007; Jouve, 2004; Smith et al., 2022). The allometric patterns recovered in the analyses were consistent with those reported by Hone et al. (2021), in which *R. muensteri* demonstrated negative allometry growth in most of the tested skeletal elements (PCRW/body, humerus, radius/ulna, metacarpal IV, and femur), isometry in the skull, neck, wing phalanx IV tail, and tibia/tarsus, and positive allometry in the wing phalanges II–III (Figure 12a). In contrast, eupterodactyloid pterosaurs exhibit positive allometric growth in most tested skeletal elements (metacarpal IV and wing phalanx I–IV), negative allometry in the femur, and isometry in the skull, humerus, and tibia/tarsus (Figure 12c). Euctenochasmatia, present a high degree of isometry (skull, PCRW/body, metacarpal IV, wing phalanges I–II, and femur), negative allometry in the humerus, radius/ulna, and wing phalanges III–IV, and positive allometry in the neck (Figure 12b).

These results suggest that *R. muensteri* exhibited highly allometric scaling patterns, indicating a reduced scaling in the PCRW/body, humerus, radius/ulna, metacarpal IV, and femur (Figure 12a). By contrast, Eupterodactyloidea underwent highly allometric scaling patterns, with a greater relative increase in size primarily in the wing elements (metacarpal IV, wing phalanx I–IV) (Figure 12c). Meanwhile, Euctenochasmatia displayed highly conservative allometric scaling (skull, PCRW/body, metacarpal IV, wing phalanges I–II, and femur) (Figure 11b).

The allometric patterns identified in our analysis suggest that *R. muensteri* had a significantly different natural history compared to the Eupterodactyloidea and Euctenochasmatia pterosaurs included in the study, particularly in traits influenced by the differential scaling of skeletal elements, such as flight style. This supports the idea that different skeletal elements exhibit varying allometric patterns in response to individual development, with some showing different allometric trajectories or even scaling independently of overall body size. The isometric pattern observed in some skeletal elements suggests a conservative scaling relationship and ontogenetic independence. This indicates that these elements may be more suitable for use in phylogenetic analysis due to their stability and consistent scaling relationship. In contrast, the allometric pattern observed in other elements may indicate ontogenetic dependence and a potential influence on phylogeny. The allometric pattern further demonstrates that the allometric pattern does not show intraspecific variation in these skeletal elements, supporting the findings of Habib and Hone (2024). Their analysis revealed lower intraspecific variation in limb proportions and other skeletal elements, with no significant osteological or proportional variability. This consistency in skeletal allometric trajectories aligns with the observed pattern of reduced intraspecific variation.

The analysis presented here yields significant results, highlighting how different traits of pterosaurs suggest distinct lifestyles across various lineages, particularly between *Rhamphorhynchus muensteri*, Eupterodactyloidea and Euctenochasmatia (Wiens, 2017; Yu et al., 2023). These differences primarily depend on whether the skeletal elements exhibit positive allometry, negative allometry, or isometry (Figure 12a–c). Such differences can influence morphological features and drive the diversification of Pterosauria. The distinct allometric trajectory of *R. muensteri* is evident when compared to the other two pterosaur groups, especially Eupterodactyloidea, due the high degree of positive allometry. The results indicate that *R. muensteri* tended to exhibit a more conservative allometric trajectory than Eupterodactyloidea and Euctenochasmatia, which could directly influence their body posture and behaviors.

The allometric pattern of the skull in the pterosaurs studied here suggests that, although variation is observed during different life stages, particularly when analyzing cranial crests (e.g., *Pteranodon longiceps*, *Nyctosaurus gracilis*, and *Dsungapterus weii*), the groups studied exhibit a uniform allometric trajectory (Figures 5a, 7a and 9a). Some authors argue that the length of the skull or rostrum may be associated with feeding specialization or weight reduction as well as differences in cranial or premaxillary crests, which could influence flight aerodynamics (Bantim et al., 2015; Brower & Veinus, 1981; Foth et al., 2012; Frey et al., 2003; Henderson, 2024; Osi, 2011; Tomkins et al., 2010; Witton & Naish, 2008). However, specialization in feeding ecology would be more closely related to differences in the morphology of the skull, mandible, and teeth, making it challenging to infer ecological variation in feeding ecology based solely on skull length (from the occipital to the tip of the rostrum). As observed by Navarro et al. (2018), skull size accounts for only 6% of the variation in shape within Pterosauria.

The positive allometry generally observed in Eupterodactyloidea forelimbs may be associated with a distinct flight style, in which wing elongation outpaces overall body growth trajectory (Figure 12c). This characteristic is consistent with obligate/extreme aerial behavior and possibly migratory habits, enabling travel across greater geographic distances (i.e., Cerqueira et al., 2021). Conversely, most forelimb skeletal elements in Euctenochasmatia exhibit negative allometry or isometry (Figure 12b). This likely limited wing elongation relative to body size, indicating a greater dependence on active flapped flight or the absence of high‐performance migratory behavior. Meanwhile, the forelimb elements of *R. muensteri* exhibit negative, positive allometry, and isometry (Figure 12a).

The distinct allometric pattern in the wing of *R. muensteri* may indicate a certain efficiency in gliding performance, allowing these animals greater migratory efficiency not necessarily over long distances but effective within the archipelago environment in which *R. muensteri* was found (Keupp et al., 2007; Tischlinger & Schweigert, 2020). The suggested increased reliance on active flight in *R. muensteri* is supported by the isometry observed in the skull, neck, tibia/tarsus, tail, and wing phalanx IV, together with negative allometry in the humerus, radius/ulna, metacarpal IV, and PCRW/body indicating a reduced anterior mass and a more posterior center of mass, an important feature for enhanced maneuverability in flight.

The positive allometry in the neck of Euctenochasmatia as well as the isometry of the PCRW/body could be associated with the foraging strategy in the shallow waters, as well a more precocial morphological pattern (Bestwick et al., 2018; Codorniú, 2007; Codorniú et al., 2018; Witton, 2013). Unfortunately, allometric analysis of the neck and PCRW/body in Eupterodactyloidea was not possible due to the absence of preserved data for most of the elements.

The observed differences in flight behavior may reflect distinct development strategies of flight, with *R. muensteri* and Euctenochasmatia potentially exhibiting a more precocial flight capability compared to Eupterodactyloidea (Hone et al., 2021; Witton & Habib, 2010). A long wing in large animals may be associated with size‐dependent differences in flight behavior, where larger pterosaurs likely exhibited slower flapping or even gliding flight. In contrast, smaller pterosaurs may have engaged in greater flapping flight, a pattern already observed in birds (Chinsamy & Elzanowski, 2001; Henderson, 2010; Palmer, 2011; Prondvai & Hone, 2008; Sato et al., 2009; Witton, 2008; Witton & Habib, 2010). According to Bennett (2015), *R. muensteri* exhibited a more rigid and inextensible wing, providing gliding flight while allowing good maneuverability due to the combination of a rigid dactylopatagium and a flexible plagiopatagium. The negative allometry of the proximal elements of the wing, together with the isometry of the distal elements, could indicate that *R. muensteri* had an early flight capability, with very young animals displaying flight behavior. This is supported by observations in bats, in which the distal elements of the wing show isometry, and juvenile bats are capable of flight at 4 or 5 weeks of age (Geipel et al., 2013; Hone et al., 2021; Naish et al., 2021; Starck & Ricklefs, 1998). The positive allometry in the mid‐wing elements could indicate an enhanced gliding capability during migratory flight, allowing more efficient use of energy throughout the journey. In contrast, the more isometric wing proportions observed in Euctenochasmatia may suggest a greater reliance on active flapping flight and consequently higher energy expenditure during locomotion.

The hindlimbs exhibit significant differences in allometric trajectories among *R. muensteri* (femur showing negative allometry, and tibia/tarsus and tail showing isometry), Euctenochasmatia (femur showing isometry), and Eupterodactyloidea (femur showing isometry and tibia/tarsus showing negative allometry) (Figure 12a–c). Unfortunately, allometric analysis of the tails of Euctenochasmatia and Eupterodactyloidea was not possible due to the shortness of data. According to Witton and Habib (2010), a dichotomy in the hindlimbs relative to forelimb length enhances flight efficiency by optimizing wing proportions while maintaining proportional stride lengths between the fore and hindlimbs during walking. The results presented here demonstrate that the allometric trajectory of the hindlimbs would influence flight in *R. muensteri* as well as in the other two groups of pterosaurs analyzed in this study. The hindlimbs of *R. muensteri* exhibit the same allometric patterns as the forelimbs, which may indicate that this species was more aerial than terrestrial (Witton, 2008).

The greater relative elongation in humerus length compared to the femur is a pattern observed in birds, especially in large‐bodied species, and has been shown to influence launch loads (Bennett, 1996; Habib, 2008; Maher et al., 2022; Padian, 2003). A similar pattern of reduced relative size of the hindlimbs compared to the forelimbs is observed in Eupterodactyloidea. This divergence in allometric trajectory between the proximal elements of both hind and forelimbs may indicate a more altricial development in Eupterodactyloidea, reflecting a lifestyle more focused on continuous flight, possibly pelagic. The isometry observed in the hindlimb of Euctenochasmatia is a typical feature of precocial species, which exhibit functionally developed hindlimbs for terrestrial locomotion from early life stages (Yang et al., 2023). This isometric pattern suggests a shallow water foraging strategy, in which the hindlimbs supported the animal on soft substrates along shorelines. The terrestrial or marginal foraging behavior of Euctenochasmatia has already been discussed by several authors, with a general consensus that their hindlimbs' skeletal structures were important for wading and providing stability on soft substrate (Bestwick et al., 2018; Hone et al., 2023; Witton, 2013).

The long tail observed in *R. muensteri*, as noted by Wellnhofer (1975), likely functioned as a stabilizing mechanism during flight, with a vane at its end providing inertial control. In contrast, pterosaurs with short tails likely exhibited reduced stability but greater maneuverability (Bramwell & Whitfield, 1974; Chatterjee & Templin, 2004). Some authors have proposed that the tail also played a role in yaw control and acted as a rudder, contributing to more aerodynamic flight (Wellnhofer, 1991; Unwin & Martill, 2003; Jagielska et al., 2024). However, Habib and Hone (2024) suggested that the long tail and vane of *R. muensteri* were more important for control and balance during ground locomotion and climbing, arguing that the tail of *R. muensteri* shares similarities with those of dromaeosaurids.

Although tails were not preserved in sufficient data for allometric analysis in Eupterodactyloidea and Euctenochasmatia, the available data for *R. muensteri* allow us to infer a distinct functional role of the tail in flight dynamics. Nevertheless, the high degree of allometry in the skeletal elements of *R. muensteri* suggests that the tail could have played a more significant role in the flight of these animals than previously thought, whether as a stabilizer, in yaw control, or even in controlling the center of mass. While the long tail would also have been important for ground locomotion, it likely served primarily as a stabilizer during flight. These differences in tail length suggest distinct flight strategies and body posture between *R. muensteri* and pterosaurs with short tail, with *R. muensteri* relying on a stabilizing mechanism for less dynamic flight activity compared to others. The findings of this study support the role of the long tail in *R. muensteri* as a stabilizer in gliding flight, with its reduced size‐related allometric trajectory correlation with that of the forelimbs, indicating a reduced reliance on flapping flight.

## 5 CONCLUSION

Our research demonstrates that *R. muensteri* exhibits significant difference in allometric patterns compared with Eupterodactyloidea and Euctenochasmatia, reflecting distinct size–shape relationships, flight strategies and paleoecological adaptations. *R. muensteri* displays a high degree of negative allometry in several skeletal elements (PCRW/body, humerus, radius/ulna, metacarpal IV, and femur) and isometry in the (skull, neck, wing phalanx IV, tail, and tibia/tarsus). These patterns suggest a flight style consistent with precocial flight capability and a reliance on active flapped flight interspersed with gliding, particularly during migratory periods, allowing the animal to conserve energy, a pattern supported by the pronounced allometric relationships among skeletal elements. The negative allometry of the PCRW/body, together with the presence of a long tail, associated with the vane, likely functioned as a stabilizing mechanism during flight, aiding in yaw control and potentially balancing of the center of mass.

Euctenochasmatia displays a high degree of isometry in the skeletal elements (skull, PCRW/body, metacarpal IV, wing phalanges I–II, and femur), and negative allometry in the humerus, radius/ulna, wing phalanges III–IV. Additionally, positive allometry is observed in the neck, suggesting a greater reliance on active flapping flight behavior and a shallow water foraging strategy. In contrast, Eupterodactyloidea demonstrated positive allometry in most of their skeletal elements analyzed (metacarpal IV, wing phalanx I–IV), while showing isometry in the skull, humerus, and femur and negative allometry in the tibia/tarsus. These patterns support a more dynamic, active flight characterized by greater maneuverability and possibly migratory habits. Additionally, the hindlimbs may have played a direct role in bearing launch loads during take‐off.

Overall, these findings emphasize the functional significance of skeletal allometry in shaping the diversity of flight behaviors and paleoecological roles within Pterosauria. The results also demonstrate that variation in skeletal allometric patterns may influence the phylogeny of the group, underscoring the importance of incorporating allometric analyses into phylogenetic studies. These findings provide new insights into the evolutionary trajectories of pterosaur anatomy, contributing to more accurate phylogenetic reconstructions.

## AUTHOR CONTRIBUTIONS

Gabriela M. Cerqueira: Conceptualization; methodology; investigation; formal analysis; visualization; writing—original draft. João L. Silva: Conceptualization; methodology; investigation; formal analysis; visualization; writing—original draft. Ingmar Werneburg: Conceptualization; investigation; writing—original draft. Mario Bronzati: Conceptualization; investigation; writing—original draft. Alex S. S. Aires: Conceptualization; writing—original draft. Felipe L. Pinheiro: Conceptualization; investigation; writing—original draft. Rodrigo T. Müller: Conceptualization; investigation; visualization; writing—original draft.

## Supporting information


Data S1.

## Data Availability

The data that supports the findings of this study are available in the supplementary material of this article.
