# Supplementary material for: Allometric patterns in Rhamphorhynchus muensteri and their role in the paleoecology, life habits, and taxonomic framework of pterosaurs
Source: J Anat. 2026 May 6:10.1111/joa.70146. Online ahead of print. doi: 10.1111/joa.70146 (PMC13645348; doi:10.1111/joa.70146)
Supplement: Supplementary file 1 [file JOA-9999-0-s001.zip › Supplementary Data 2.docx]

**Supplementary Information**

Contribution of (in percentage) of each skeletal variable to the principal components 1 and 2, resulted from the PCA performed to Eupterodactyloidea. Abbreviations: PCRW/Body, precaudal trunk spine; McIV, metacarpal IV.

| **Variables** | **PC1** | **PC2** |
| --- | --- | --- |
| Skull | 7.53 | 65.43 |
| Humerus | 10.52 | 0.18 |
| Radius/Ulna | 10.63 | 0.02 |
| McIV | 9.99 | 13.35 |
| Wing phalange I | 10.18 | 9.91 |
| Wing phalange II | 10.50 | 2.84 |
| Wing phalange III | 10.39 | 2.53 |
| Wing phalange IV | 9.70 | 2.51 |
| Femur | 10.37 | 2.72 |
| Tibia/Tarsus | 10.13 | 0.46 |

Contribution of (in percentage) of each skeletal variable to the principal components 1 and 2, resulted from the PCA performed to Euctenochasmatia. Abbreviations: PCRW/Body, precaudal trunk spine; McIV, metacarpal IV.

| **Variables** | **PC1** | **PC2** |
| --- | --- | --- |
| Skull | 8.96 | 0.46 |
| Neck | 6.63 | 0.60 |
| PCRW/body | 0.44 | 97.23 |
| Humerus | 9.06 | 0.16 |
| Radius/Ulna | 9.17 | 0.37 |
| McIV | 9.01 | 0.18 |
| Wing phalange I | 9.09 | 0.09 |
| Wing phalange II | 9.23 | 0.03 |
| Wing phalange III | 9.13 | 0.16 |
| Wing phalange IV | 8.97 | 0.01 |
| Femur | 9.01 | 0.38 |
| Tibia/Tarsus | 9.23 | 0.28 |

Contribution of (in percentage) of each skeletal variable to the principal components 1 and 2, resulted from the PCA performed to *Rhamphorhynchus muensteri*. Abbreviations: PCRW/Body, precaudal trunk spine; McIV, metacarpal IV.

| **Variables** | **PC1** | **PC2** |
| --- | --- | --- |
| Skull | 7.78 | 1.2 |
| Neck | 7.52 | 3.6 |
| PCRW/body | 7.79 | 4.9 |
| Humerus | 6.98 | 50.2 |
| Radius/Ulna | 7.85 | 0.12 |
| McIV | 7.30 | 15.8 |
| Wing phalange I | 7.93 | 1.2 |
| Wing phalange II | 7.88 | 4.5 |
| Wing phalange III | 7.75 | 9.6 |
| Wing phalange IV | 7.75 | 0.001 |
| Tail | 7.83 | 0.13 |
| Femur | 7.78 | 0.18 |
| Tibia/Tarsus | 7.79 | 0.6 |

PCA plots refer to Eupterodactyloidea pterosaurs. (a), percentage of explained variance of dimensions; (b), contribution of the ten variables, which contributes more to PC1; (c), Contribution of the ten variables, which contributes more to PC2. Abbreviations: McIV, metacarpal IV; Ph I-IV, wing phalanx I-IV.

PCA plots refer to Euctenochasmatia pterosaurs. (a), percentage of explained variance of dimensions; (b), contribution of the ten variables, which contributes more to PC1; (c), Contribution of the ten variables, which contributes more to PC2. Abbreviations: McIV, metacarpal IV; Ph I-IV, wing phalanx I-IV.

PCA plots refer to *Rhamphorhynchus muensteri* pterosaurs. (a), percentage of explained variance of dimensions; (b), contribution of the ten variables, which contributes more to PC1; (c), Contribution of the ten variables, which contributes more to PC2. Abbreviations: McIV, metacarpal IV; Ph I-IV, wing phalanx I-IV.
